# Knee Angle Estimation from Surface EMG during Walking Using Attention-Based Deep Recurrent Neural Networks: Feasibility and Initial Demonstration in Cerebral Palsy

**DOI:** 10.3390/s24134217

**Published:** 2024-06-28

**Authors:** Mohamed Abdelhady, Diane L. Damiano, Thomas C. Bulea

**Affiliations:** Rehabilitation Medicine Department, National Institutes of Health Clinical Center, Bethesda, MD 20892, USA; mohamed.abdelhadi@nih.gov (M.A.); damianod@cc.nih.gov (D.L.D.)

**Keywords:** electromyography, deep learning, estimation, cerebral palsy

## Abstract

Accurately estimating knee joint angle during walking from surface electromyography (sEMG) signals can enable more natural control of wearable robotics like exoskeletons. However, challenges exist due to variability across individuals and sessions. This study evaluates an attention-based deep recurrent neural network combining gated recurrent units (GRUs) and an attention mechanism (AM) for knee angle estimation. Three experiments were conducted. First, the GRU-AM model was tested on four healthy adolescents, demonstrating improved estimation compared to GRU alone. A sensitivity analysis revealed that the key contributing muscles were the knee flexor and extensors, highlighting the ability of the AM to focus on the most salient inputs. Second, transfer learning was shown by pretraining the model on an open source dataset before additional training and testing on the four adolescents. Third, the model was progressively adapted over three sessions for one child with cerebral palsy (CP). The GRU-AM model demonstrated robust knee angle estimation across participants with healthy participants (mean RMSE 7 degrees) and participants with CP (RMSE 37 degrees). Further, estimation accuracy improved by 14 degrees on average across successive sessions of walking in the child with CP. These results demonstrate the feasibility of using attention-based deep networks for joint angle estimation in adolescents and clinical populations and support their further development for deployment in wearable robotics.

## 1. Introduction

Surface electromyography (EMG) reflects the electrical activity generated within contracting muscle fibers in response to activation by the nervous system. Surface EMG (sEMG) signals can be collected by electrodes attached to the skin over the target muscle. These signals contain information about the neural drive (i.e., the number and intensity of the activated motor neurons) and the fiber electrical properties [1]. Thus, when collected during volitional movement, sEMG encodes information about movement intent, which makes it suitable for use as a control signal for human–machine interfaces, wearable robotics, and exoskeletons [2,3]. Measurable changes in sEMG occur 20–300 ms earlier than the muscle contraction and resulting movement [1,4]. Therefore, sEMG offers the potential benefit of earlier detection of human motion intention compared to kinematic signals like limb movement, joint angle and angular velocity. This potential makes sEMG particularly attractive for real-time adaptive systems that adjust performance based on user intent. EMG signals have been used in a myriad of ways for action classification and torque and angle estimation [2]. In wearable robotics, joint angle estimation is key for achieving smooth motion control and has broad application prospects [5].

Yet, estimating knee angle at different walking speeds using sEMG is challenging due to various factors that affect the EMG signals, such as muscle contraction level, movement speed, and individual gait pattern, among others. The relationship between EMG and joint angle varies with walking speed, which can impact its ability to estimate joint angles [6]. For instance, sEMG may be more reliable for estimating joint angles during slower walking speeds but may become less reliable as speed increases [7]. Also, different muscles may play a more or less significant role in controlling joint movement at different walking speeds. For example, in slow walking, the quadriceps may be more important in controlling—and therefore, estimating—knee movement, whereas during fast walking, the hamstrings may play a greater role [8]. An additional challenge is variability across individuals, including motor control strategy, gait patterns, and age. As people age, changes occur in the neuromuscular system that affect the relationship between sEMG and joint angle. For example, older adults may have weaker muscles, slower activation times, and altered recruitment patterns, which can make accurate estimation of joint angles from sEMG difficult. Additionally, older adults may have different gait patterns than younger adults, which can further complicate knee angle estimation [9]. Children and adolescents also pose challenges. The neuromuscular system undergoes significant changes during development, which results in increased stride-to-stride variability in EMG during walking compared to adults [9]. Further, although a stable kinematic gait pattern is typically achieved by age 8 years, motor control and associated muscle activation patterns continue to develop beyond this age. The musculoskeletal system also continues to develop through adolescence, which can affect the relationship between muscle activation and joint movement patterns and make it more difficult to estimate joint angles accurately from sEMG alone.

Estimating joint angles from sEMG typically involves four steps: signal acquisition, signal processing, feature extraction, and model selection [10]. This study focuses on feature extraction and model selection for the purposes of estimating knee angle during walking, with the ultimate goal of creating novel approaches to controlling a wearable knee exoskeleton. Features can be classified into time-domain, frequency-domain, and time-frequency-domain features, with time-domain features being the most commonly used [5,11]. A variety of techniques to improve the accuracy of kinematic estimation from sEMG-based features have been developed, such as using machine learning algorithms to account for individual variability and differences across movement tasks [12,13,14,15,16].

The models deployed to estimate joint angles from sEMG can be broadly classified into three main categories. The first category deploys the Hill muscular model which necessitates the understanding of muscle–tendon velocity and length as well as other variables (e.g., velocity of contraction, and coefficient of shortening heat) that are likely to vary between persons [17,18] and are therefore difficult to determine a priori. As a result, the performance of the Hill muscular model for joint angle estimation may be inaccurate [19]. The second general approach to estimating kinetics from EMG signals involves data-driven techniques such as principal component analysis [20] and non-negative matrix factorization (NMF) [21]. Unlike physics-based models that require extensive measurements and computations, data-driven approaches offer a more straightforward and faster solution. PCA has shown effectiveness in reducing the computational burden of EMG signals, while NMF has been utilized to explore both upper and lower body kinematics [22,23]. These algorithms extract essential features and patterns from the EMG data, allowing for efficient estimation of kinetics and kinematics without relying on complex physical models [22].

The third category of joint angle estimation using EMG, machine learning (ML) methods, has gained significant prominence in recent years [24,25]. This category of methods encompasses approaches that take sEMG as inputs and output kinematic estimates without deploying an underlying canonical model of biomechanics. ML algorithms have demonstrated promising results in capturing the intricate relationships between EMG signals and joint angles, enabling more precise and reliable predictions. The application of machine learning to estimate kinetic and kinematic information from EMG signals has two main applications. The first one focuses on predicting human activity and intent using machine learning classifiers [26]. By analyzing EMG signals, these classifiers can discern different human activities to facilitate natural human–computer interactions and human–robot interactions. The second application employs regression techniques with machine learning to estimate kinetic and kinematic parameters of movement [27]. Leveraging EMG data, regression models predict joint angles, muscle forces, or other biomechanical variables, such as lower limb joint angles during walking. There are a multitude of ways in which machine learning approaches have been formulated to estimate joint angles during walking. For example, Li et al. [27] proposed a least squares support vector regression (LS-SVR) algorithm to estimate the joint angle in the lower limb using multi-channel sEMG signals. LS-SVR is an improved version of support vector machine (SVM) that addresses high computational costs. The LS-SVR inputs include the integral absolute value (IAV) features of seven lower limb muscles. Xiao et al. [28] used random forests (RF) with multiple time-delay features to estimate the joint angle of the human lower limb with an average root mean square difference value of 3.11 ± 0.4 degrees. Zhang et al. [29] developed an angle prediction model using a backpropagation neural network with the root mean square (RMS) feature of sEMG signals to describe the relationship between leg joint angles (hip, knee or ankle) and sEMG signals. The average RMS error (RMSE) of their model for knee extension was 9 degrees.

Li et al. [30] proposed a wavelet neural network (WNN) model for knee-joint angle estimation from sEMG and showed that it outperformed the radial basis function (RBF) neural network and the backpropagation (BP) neural network models when estimating knee angle from sEMG. This was unexpected as the WNN model is a relatively new approach and has not been extensively used in this domain before. However, the study only included one able-bodied person, so further research is needed to confirm these findings on a larger and more diverse population. Tahamipour-Z. et al. used a multilayer perceptron artificial neural network (MLPANN) to estimate knee torques from sEMG signals and found that the MLPANN performed better than other neural network models [31]. Taken together, these studies highlight the potential of deep neural networks in accurately estimating knee joint kinematics using sEMG signals.

In recent years, deep learning algorithms, particularly recurrent neural networks (RNNs) and long short-term memory (LSTM) networks, have gained prominence for joint angle estimation based on surface electromyography (sEMG) signals. Huang [32] effectively utilized RNN to estimate real-time knee joint angles, leveraging the sequence nature of sEMG data. LSTM networks, known for their ability to handle longer sequences, have also been employed in this context. Rezaie Zangene et al. and Sohane and Agarwal [33,34] utilized LSTM networks to predict knee joint kinematics from sEMG signals, with Rezaie Zangene et al. [33] finding superior prediction accuracy compared to conventional fully connected neural networks. Meanwhile, to address the gradient vanishing issue in RNNs with longer sequences, LSTM was introduced and has been employed by researchers like Wang [35] and Chen [4] for joint angle prediction in different studies. Additionally, Huang et al. [32] introduced deep-recurrent neural networks (DRNNs) and wavelet neural networks (WNNs) for real-time knee-joint angle prediction using sEMG and inertial measurements, highlighting the effectiveness of these deep learning approaches. Similarly, Zangene et al. [33] proposed an innovative method using sEMG and LSTM networks to estimate knee and ankle joint kinematics during squat training, with LSTM-based sequence-to-sequence models outperforming conventional fully connected neural networks in terms of prediction accuracy. Recently, Zangene et al. presented a deep learning model called AM-BiLSTM, integrating attention mechanism and bidirectional LSTM network, to estimate knee joint angle accurately during running at different speeds using minimal sEMG sensors [36]. The proposed AM-BiLSTM model’s generalization ability was evaluated in various scenarios, including different participants, speeds, and combinations thereof. It outperformed BiLSTM, standard LSTM, and MLP techniques, showing significantly better performance with lower normalized root mean square error and higher correlation coefficient values (*p*-value < 0.05) [36].

Whereas clear evidence exists for the effective use of ML and deep learning models for joint estimation from sEMG, consideration of intra- and inter-participant variability has been relatively limited with the majority of studies focusing on a small number of participants or even a single individual, which may not capture the full range of EMG patterns and movements that exist within and across diverse populations. Each person’s EMG patterns can be influenced by various factors, including their anatomy, physiology, age, and even their specific movement habits. As such, it becomes imperative to account for participant-specific characteristics in the estimation process. By incorporating a broader and more diverse range of participants, researchers can better understand the variability in EMG signals and design more robust and adaptable estimation algorithms. Similarly, most studies have completed evaluations with data from a single visit, but significant potential sources of variability arise across days, such as electrode placement, the electrode–skin interface, and even movement patterns, presenting a significant challenge in terms of model training. This is particularly important if EMG data are intended for the control or evaluation of exoskeletons across multiple days. Thus, ensuring accurate and consistent joint estimation from muscle activity patterns over time is crucial for efficient and effective exoskeleton usage. Addressing visit-to-visit variability adds another layer of complexity, necessitating comprehensive research into how EMG signals might fluctuate within and between participants during different sessions. A holistic approach that encompasses both intra- and inter-participant variability will contribute to the development of more robust and reliable strategies for utilizing EMG signals in the realm of exoskeleton control and assessment.

Another noteworthy limitation in previous studies is the lack of comparison between the estimation accuracy of non-healthy participants and healthy individuals. While many researchers focus on studying healthy participants, if the ultimate goal is the use of estimation algorithms in individuals with motor limitations, their inclusion in studies evaluating the estimation of joint angles from sEMG during coordinated movements such as walking is critical to understanding the generalizability of these methods. Bridging this gap would lead to more inclusive and practical solutions, allowing for the development of assistive technologies and robotic systems that can cater to a diverse range of users, regardless of their health status.

Attention mechanisms that prioritize the use of the most predictive inputs can be deployed to improve the estimation accuracy of deep learning algorithms, offering a potential method to reduce the effect of variability across individuals and sessions within individuals. Indeed, such combined approaches have been previously shown to be effective in estimating finger kinematics from sEMG [37] and in predicting upper limb movements from sEMG in individuals post-stroke [38]. Here, we propose to predict knee angle from sEMG during walking by combining an attention mechanism built on an encoder–decoder approach in neural networks with a gated recurrent unit (GRU) neural network, an updated version of LSTM suitable for time series data that mitigates the vanishing gradient problem found in RNNs [39]. Despite being an active area of research in recent years, as evidenced by the above studies, the practicality and robustness of these methods and their application in particular to reliably estimate joint angles in real-world scenarios, remains unknown.

Our overall objective is to develop an estimator for real-time prediction of the knee angle from sEMG during exoskeleton-assisted walking in individuals with neuromotor disorders. This study presents our preliminary effort to achieve that goal through three successive experiments. First, we evaluate the ability of a GRU to estimate knee joint angle during overground walking from eight channels of sEMG data recorded from four adolescents with typical development, with and without the attention mechanism (AM), to confirm its inclusion enhanced performance accuracy. We supplemented our analysis by performing a sensitivity analysis to assess the impact of each individual sEMG channel on the GRU-AM performance. The formulation of the GRU and AM for this application and the results of its knee angle prediction accuracy and sensitivity were previously presented in a conference abstract [40]. To ensure completeness and facilitate understanding of the additional experiments, we present the GRU-AM prediction model development and its initial application here as Experiment 1. Then, we extend these findings and introduce novel and important extensions of this work through additional experiments and analysis as follows. In the second experiment, we evaluate the transfer learning capability of the GRU-AM model by first training it on an open-source data set [41] before training again on the same data set as the first experiment (four typically developing adolescents) and then assessing its predictive performance compared to the first experiment. We hypothesize that given its ability to identify the input elements that are most critical to accurate prediction, combined with the fact that walking (at least in healthy populations) is similar across individuals, the GRU-AM will be effective when trained on one set of data and applied to data from a different person. Finally, given our overall goal, we perform a third experiment in which the GRU-AM model that was pretrained on the open source dataset is applied to estimate knee joint angle from sEMG data recorded from one individual with cerebral palsy (CP) on three successive visits. Here, we hypothesize that the GRU-AM will result in continuously improving prediction across sessions because the most salient input features for prediction should not vary across days.

The main contributions of this manuscript compared to our and other prior work are to evaluate the potential real-world and clinical viability of deploying the GRU-AM estimation method to estimate lower limb kinematics from sEMG. Specifically, we evaluate the utility of transfer learning to enhance GRU-AM model prediction accuracy by examining the performance of a previously trained model applied to new individuals. Further, we examine for the first time the ability of a GRU-AM to estimate knee joint angle in an individual with a neuromotor disorder (CP) walking overground while wearing an exoskeleton. Finally, we extend the clinical analysis by combining it with the transfer learning process within an individual to examine the ability of the GRU-AM to estimate knee angle across multiple visits, which represents a true clinical workflow. Collectively, these results will provide insight into the ability of the GRU-AM estimator to function in real-world scenarios, including transfer between individuals and day-to-day use within the same individuals and within a clinical context.

## 2. Methods

### 2.1. Gated Recurrent Unit with Attention Mechanism

**Figure 1:**
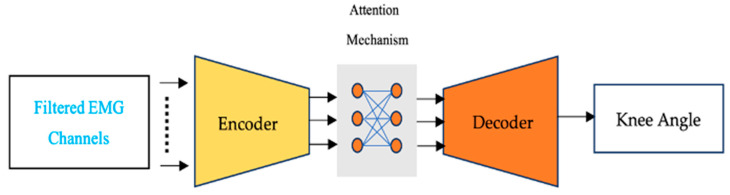
A block diagram representing the pipeline to estimate joint angle during walking from sEMG using an attention mechanism.

Attention mechanisms, which are advancements of encoder–decoder models, have emerged as one of the frontiers in deep learning for improving the performance of processing long input sequences [42]. The attention mechanism assigns a weight or importance score to each input element, based on its relevance to the current system output. This allows the model to selectively focus on the most salient parts of the input and ignore irrelevant or noisy information. The attention mechanism involves three main components: a query, a set of key–value pairs, and a scoring function. The query is a vector that represents the current context or state of the model, while the key–value pairs represent the input data. The scoring function computes a similarity score between the query and each of the key–value pairs, which is used to compute a weight or importance score for each value. The values are then combined using a weighted sum, where the weights are the importance scores computed by the attention mechanism. The attention mechanism can be integrated into various neural networks, including RNNs, convolutional neural networks (CNNs), and transformer models. The transformer/attention mechanism model (Figure 1), which was introduced first for natural language processing, is a popular and powerful model architecture that deploys the attention mechanism to achieve excellent performance on a range of tasks [43].

The decoder in attention mechanisms can selectively access the encoded information, using the context vector c(t), which is computed at each time step of the decoder based on the previous hidden state and all the hidden states of the encoder. The relative importance of each element in the input sequence is determined by trainable weights assigned to these states, with greater emphasis placed on the most significant inputs, giving rise to the name “attention mechanisms” [44]. The context vector is constructed by combining each time step j of the encoder with each time step t of the decoder, and the alignment score. The formula for computing the alignment score is given as:

(1) $$score\left(j,t\right)=V_{a}tanh(U_{a}s\left(t-1\right)+W_{a}h\left(j\right))$$

where $V_{a}$, $W_{a}$, and $U_{a}$ are the trainable weights that comprise the attention mechanism. $V_{a}$ weights the alignment score, while $U_{a}$ and $W_{a}$ are associated with the hidden states of the decoder (s(t)) and encoder (h(j)), respectively. To obtain the attention weights α(j,t) for each time step t, the alignment score must be normalized. This is achieved using the SoftMax function in conjunction with the time steps j, and the resulting attention weights are defined as follows:

(2) $$\alpha \left(j,t\right)=\frac{e^{score\left(j,t\right)}}{\sum_{j=1}^{M}e^{score\left(j,t\right)}}$$

The attention weights enable the trainable weights to capture the relative importance of the input at time step j in decoding the output at time step *t*. Once the attention weights are determined, the context vector is computed as the weighted sum of the relationship between all the encoder hidden values h(i) and the attention weights. This relationship is given by:

(3) $$c\left(t\right)=\sum_{j}\alpha \left(j,t\right)h(j)\;$$

By incorporating the context vector into the decoding process, attention mechanisms enable more focus on the relevant inputs in the EMG features. The context vector c(t) is passed through the decoder to calculate the probability for the next possible output, which is performed for all time steps at the input. After this step, the current hidden state s(t) is computed based on the context vector c(t), the previous hidden state s(t−1), and the output $\hat{y}(t-1)$ from the previous time step as follows:

(4) $$s\left(t\right)=f(s\left(t-1\right),\hat{y}\left(t-1\right),c\left(t\right))$$

The attention mechanism described above allows the model to identify the correlations between different parts of the input sequence and the corresponding parts of the output sequence. At each time step, the decoder output is computed using the SoftMax function applied to the hidden state, which is in turn computed using the context vector, previous hidden state, and previous output. This approach enables the model to selectively focus on the most relevant parts of the input sequence, leading to improved performance in generating the corresponding output sequence [45].

A GRU is a type of RNN used for sequential data processing. It consists of a hidden state that captures information from previous time steps. Two critical components, the update gate and the reset gate, regulate how information is retained or discarded in the hidden state. The update gate determines how much of the previous hidden state should be preserved, allowing the network to adapt its memory usage dynamically. Simultaneously, the reset gate controls the extent to which the previous hidden state is reset, enabling the GRU to respond to changing patterns in the data. The candidate hidden state, generated using the current input and the previous hidden state, is blended with the previous hidden state according to the update gate, producing the new hidden state or output. This design helps the GRU capture long-range dependencies in sequential data while addressing the vanishing gradient problem, making it suitable for various applications like natural language processing and time series analysis [46].

**Figure 2:**
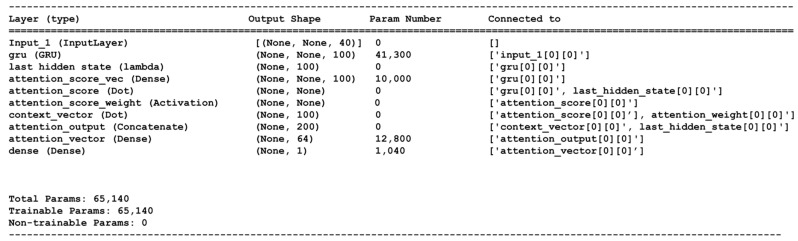
Details of the shape and layer connections in the attention-based neural network for the 8-muscle case.

In our study, we employ an encoder–decoder architecture with an attention mechanism. Specifically, the encoder–decoder units consist of 100 gated recurrent unit (GRU) cells, structured as illustrated in Figure 2. To enhance this architecture, we incorporate a precisely defined attention mechanism known as the Bahdanau attention mechanism [47]. This integration results in the creation of our model, the GRU with an attention-based mechanism (GRU-AM). To evaluate the performance of the GRU-AM estimator model, the root mean squared error (RMSE) and correlation coefficient (CC) are used as a performance metrics. RMSE is computed as

(5) $$RMSE=\sqrt{\frac{1}{N}\sum_{j=1}^{N}{\left({\tilde{\theta }}_{i}-\theta_{i}\right)}^{2}}$$

where N is the number of data points in the time series to be estimated, $\theta_{i}$ is the time series’ measured value, and ${\tilde{\theta }}_{i}$ is the prediction model’s estimated value at sampling time i. CC is calculated as

(6) $$CC=\frac{\frac{1}{N}\sum_{i=1}^{N}\left(\theta_{i}-\bar{\theta })(\tilde{\theta }-\bar{\tilde{\theta }}\right)}{\sqrt{\frac{1}{N}\sum_{i=1}^{N}{\left(\theta_{i}-\bar{\theta }\right)}^{2}}\sqrt{\frac{1}{N}\sum_{i=1}^{N}{\left(\tilde{\theta }-\bar{\tilde{\theta }}\right)}^{2}}}$$

where $\bar{\theta }$, and $\bar{\tilde{\theta }}$ is the mean of the measured and estimated knee angle vectors, respectively. To further gauge estimation quality, the signal-to-noise ratio (SNR) of the estimate knee angle serves as an additional metric and is calculated as

(7) $$SNR=10\log_{10}\left(\frac{\theta^{2}}{{\tilde{\theta }}^{2}}\right)$$

### 2.2. Deep Learning Attention Model for EMG Analysis

Each EMG signal corresponding to a single gait cycle was partitioned into five segments, each consisting of 20 samples. This resulted in a total of 40 segments for the 8-muscle input model and 30 segments for the 6-muscle channel model. We used MATLAB R2022b software to preprocess and segment the data. The deep learning attention model was created using the Keras library in Python 3.11, and its summary is shown in Figure 2. An ADAM optimization algorithm and binary cross-entropy loss function were used to train the model. The dataset was split into 60% for training and 40% for testing.

Regarding the training session of the attention-based mechanism, the session concludes either upon reaching an RMSE of less than 15 degrees or after a maximum of 10,000 epochs. Notably, attempts to enhance the termination criteria by relaxing the RMSE threshold to less than 20 or by increasing the number of epochs yielded insignificant improvements in performance. This underscores the importance of achieving a balance between accuracy and computational efficiency in the training process.

### 2.3. Data Collection and Pre-Processing

#### 2.3.1. Data Collection

In this research, three distinct datasets were employed to train and validate the GRU-AM for knee angle estimation. The first and the third datasets were collected through experimental overground trials conducted at the National Institutes of Health Neurorehabilitation and Biomechanics Research Section motion capture laboratory. In the first, 4 healthy volunteers (1 male, 3 females; age: 14.9 ± 1.1 years; height: 164 ± 7 cm; weight: 73 ± 18 kg) traversed a 5.5 m walkway repeatedly at a self-selected pace while wearing shoes. The study was approved by the NIH institutional review board and informed assent and consent were obtained from the participants. Only sEMG from steady-state walking was retained for analysis. EMG was recorded (Delsys, Trigno Wireless, Boston, MA, USA) at 1000 Hz bilaterally from the tibialis anterior (TA), medial gastrocnemius (MG), soleus (SL), peroneus longus (PL), hallucis longus (HL), rectus femoris (RF), vastus lateralis (VL), and medial hamstrings (MH). Kinematic data were recorded at 100 Hz using motion capture (Vicon, Denver, CO, USA). In the second protocol, data from one participant with CP (age: 7 years; height: 111 cm; weight: 18.5 kg) walking overground with a wearable robotic exoskeleton were used. The exoskeleton design and its control system have been presented previously [48]. The exoskeleton included a passive ankle joint and an actuator at the knee. In all data included here, the exoskeleton was operating only to compensate for its own inertia, whereby no assistive torques were provided to the limb and the exoskeleton controller enforced a net zero torque about its knee joint axis [48]. These data are part of a larger ongoing study to evaluate the effects of robotic exoskeleton assistance on children with crouch gait. The study was approved by the NIH institutional review board and informed assent and consent were obtained from the participant and his legal guardian prior to participation. The participant met all inclusion criteria for the study: age above 5 years, ability to understand and follow simple directions, less than 5 degrees of knee flexion contracture, less than 10 degrees of ankle plantar flexion contracture, measured foot–thigh angle between −10 and 25 degrees, the ability to walk at least 10 feet without stopping, and a diagnosis of crouch gait from CP.

The participant had spastic diplegic CP and was assessed as Gross Motor Function Classification System (GMFCS) [49] Level III, meaning a walking aid was required during overground walking. Like the healthy volunteers, the participant with CP walked overground across a 5.5 m walkway at self-selected pace. In the exoskeleton experiment, sEMG was recorded at 2000 Hz bilaterally from tibialis anterior (TA), medial gastrocnemius (MG), vastus lateralis (VL), rectus femoris (RF), and semitendinosus (ST). Custom pockets in the exoskeleton were created to accommodate the sEMG electrodes and prevent interference during recording. Motion capture data were recorded at 100 Hz using a custom marker set placed on the pelvis, thighs, shanks and feet [48].

The second dataset utilized in the study is an open-source dataset encompassing locomotion trials performed by healthy participants across four diverse terrain types: treadmill, level ground, ramp, and stairs [41]. During the experiments, the participants were equipped with wearable sensors. Motion capture technology and force plates were used to capture comprehensive motion and force data. To process the motion capture data, the researchers utilized their custom MATLAB toolbox, MoCap Tools, which incorporates a gap-filling method to address missing data and provides a user-friendly application programming interface (API) to interface with C3D files and Opensim [41]. The same number of participants from this open-source data set (four) was randomly selected for use in Experiment 2.

#### 2.3.2. Preprocessing

**Figure 3:**
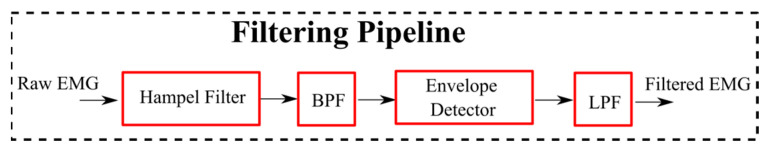
A block diagram representing the pipeline of filtering EMG signals used with healthy and CP participants.

**Figure 4:**
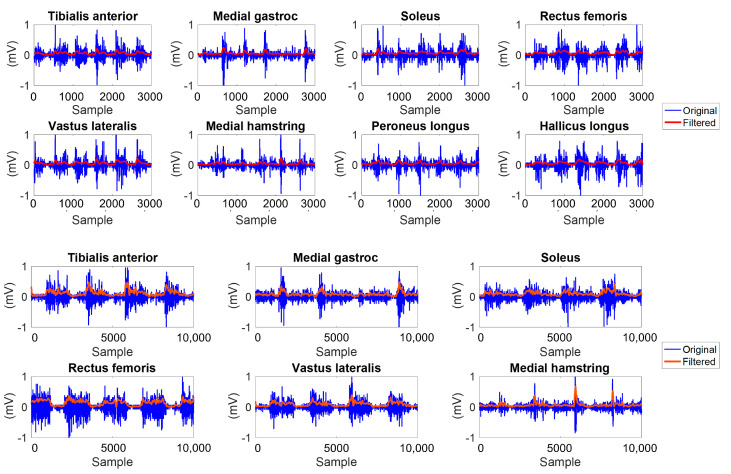
Electromyography signals from lower limb muscles were processed with multistage filtering. The top two rows exhibit recordings of 8 muscle signals acquired from a healthy participant in the NIH dataset. In contrast, the bottom two rows display electromyography signals from 6 muscles obtained from the participant diagnosed with cerebral palsy.

For both datasets collected at the NIH, kinematics data were processed offline using Visual 3D software V2022.12 (C-Motion, Germantown, MD, USA) to compute lower limb joint angles. Kinematic data from the foot markers were used to segment the walking data into gait cycles for each limb spanning from one foot-ground contact event to the next same foot contact. A multiple-stage filtration procedure was applied to process the raw EMG signals as depicted in Figure 3. The first stage involved applying a Hampel filter with a window size of 20 samples to remove any outliers present in the raw EMG signals, effectively reducing noise and artifacts. Subsequently, a bandpass filter was implemented, with cutoff frequencies set at 50 Hz and 190 Hz to pass EMG signal components within the desired frequency range, effectively removing unwanted noise and irrelevant frequencies that may have been captured during data acquisition. The next step involved rectification of the filtered EMG signal using a sliding window of 50 samples. Consecutive peak and root mean square (RMS) envelop detectors were then applied to the rectified signal to emphasize the amplitude and temporal characteristics of the EMG signal, enabling a more precise analysis of muscle activation patterns. To further refine the output, a low-pass filter was applied to smooth the filtered EMG data and remove any remaining high-frequency noise. The parameters of the multi-stage filter, such as window sizes and cutoff frequencies, were iteratively adjusted until arriving at the final values to ensure the filtration procedure was consistently and accurately performed on both datasets. An example of the output of the filtering process on the raw EMG data is shown in Figure 4.

### 2.4. Experimental Design

#### 2.4.1. Experiment 1: Comparative Analysis and Sensitivity Study

The first experiment consisted of two parts conducted using data collected from walking with four typically developing adolescents. In the first part, the study estimated knee joint angles during normal walking using a GRU with eight channels of surface electromyography (sEMG) data: tibialis anterior (TA), medial gastrocnemius (MG), soleus (SL), peroneus longus (PL), hallucis longus (HL), rectus femoris (RF), vastus lateralis (VL), and medial hamstrings (MH). EMG data were collected bilaterally; however, only the right (dominant) side is examined here. The study compared the predictive performance of GRU alone with GRU enhanced by an attention mechanism (GRU-AM). In the second part, a sensitivity analysis was conducted to determine the individual contributions of each muscle to the GRU-AM estimation. In this test, the model was evaluated by sequentially removing the sEMG channels and computing the resulting RMSE and CC.

#### 2.4.2. Experiment 2: Transfer Learning

The objective of the second experiment was to evaluate the potential of transfer learning, or the ability to utilize the information learned during one task to improve performance on a new, related task, to enhance the prediction accuracy of the GRU-AM model. Initially, the GRU-AM model was trained using sEMG data from an open source dataset [41]. Subsequently, this pre-trained GRU-AM model was assessed using the same dataset from Experiment 1. The open source dataset contains six shared sEMG channels with the dataset from Experiment 1: tibialis anterior (TA), medial gastrocnemius (MG), soleus (SL), vastus lateralis (VL), rectus femoris (RF), and medial hamstring (MH). Thus, the GRU-AM estimated knee angle from these six channels only.

The GRU-AM model was initially trained using data from four healthy participants from the open-source set, chosen at random, to match the number of participants in the original dataset of Experiment 1. Each participant contributed a minimum of three individual trials, with each trial comprising 10 consecutive strides. The data were portioned into a ratio of 60% for training and 40% for testing.

Then, to simulate the scenario of applying the pretrained model to new healthy individuals, a sequential training approach was employed across the four healthy adolescents from Experiment 1, with a training interval of three strides. The procedure was performed as follows:

1. Initial training: The GRU-AM model was trained using all trials from the four healthy participants in the open-source dataset.
2. Usage by NIH Subject No. 1: The GRU-AM model was further trained with one trial of subject-specific data involving the same number of strides as used in the open-source dataset participants’ trials (10 strides). Subsequently, the remaining data from each individual were used for knee angle estimation, with sequential training conducted every 3 strides. The evaluation of this process included monitoring the CC and RMSE for each stride.
3. The same procedure as Step 2, but applied to NIH Subject No. 2 after the initial training in Step 1.
4. The same procedure as Step 2, but applied to NIH Subject No. 3 after the initial training in Step 1.
5. The same procedure as Step 2, but applied to NIH Subject No. 4 after the initial training in Step 1.

#### 2.4.3. Experiment 3: Progressive Adaptation in a Participant with CP

The goal of this experiment was to evaluate the ability of the GRU-AM model to estimate knee joint angle during walking in a child with CP across a series of consecutive visits, which occurred approximately once every two weeks. The GRU-AM model was pretrained using data from healthy participants from the open source and NIH datasets, including four participants from each dataset. The model was initially trained using the open-source dataset, then progressively trained using each participant from the NIH dataset, and finally was tested in each of three sequential visits from the participant with CP.

Similar to Experiment 2, a sequential learning policy was employed to continually update the GRU-AM model after every 3 strides, employing the sEMG data from the participant with CP (see Data Collection section below). This experiment was designed to mimic real-world rehabilitation sessions, where the trained estimator is utilized to predict the knee angles of a participant with CP wearing a lower-limb exoskeleton. The data used for testing and sequentially training the GRU-AM model originated from the participant’s first, second, and third visits for an ongoing study.

## 3. Results

### 3.1. Experiment 1: Comparative Analysis and Sensitivity Study

**Figure 5:**
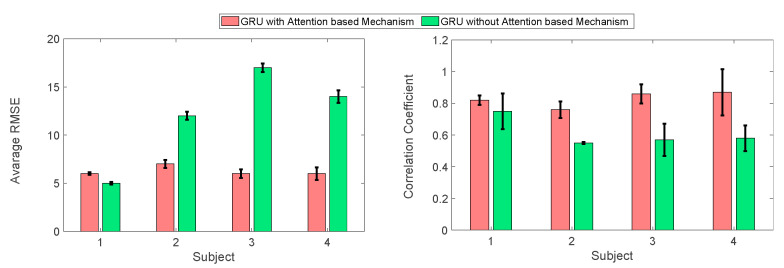
The Impact of attention-based mechanism on knee angle estimation. The performance GRU neural network with and without attention mechanism in the four healthy participants as indicated by average RMSE (degrees, **left**) and correlation coefficient (**right**). Note: these results were calculated using the testing data.

We conducted a comparative analysis of our proposed knee angle estimation model in two configurations: one with the attention mechanism (GRU-AM) and the other without it (GRU). The GRU-AM model exhibited an average model accuracy of 7.0 degrees based on the RMSE, surpassing the GRU model, which achieved an accuracy of 11.1 degrees. This notable improvement in accuracy is visually evident in Figure 5, where the GRU-AM exhibits a consistently reduced average RMSE when estimating knee joint angles compared to the GRU model without the attention mechanism. In addition to the improvements in RMSE compared to GRU, the GRU-AM performance is marked by more consistent values across different participants, suggesting that the attention mechanism was able to better leverage the most salient inputs for each individual. In stark contrast, the GRU model, when employed alone, displays significant RMSE variation from one participant to another, indicating less stability in its predictions. Further examination of the performance metrics unveils additional nuances. The GRU-AM estimator not only exhibits less RMSE variation but also shows an increase in CC across participants (0.80 ± 0.02) compared to GRU alone which is (0.68 ± 0.01). This signifies that the GRU-AM model maintains a higher degree of consistency and precision in knee angle estimation when compared to the GRU model operating in isolation.

**Figure 6:**
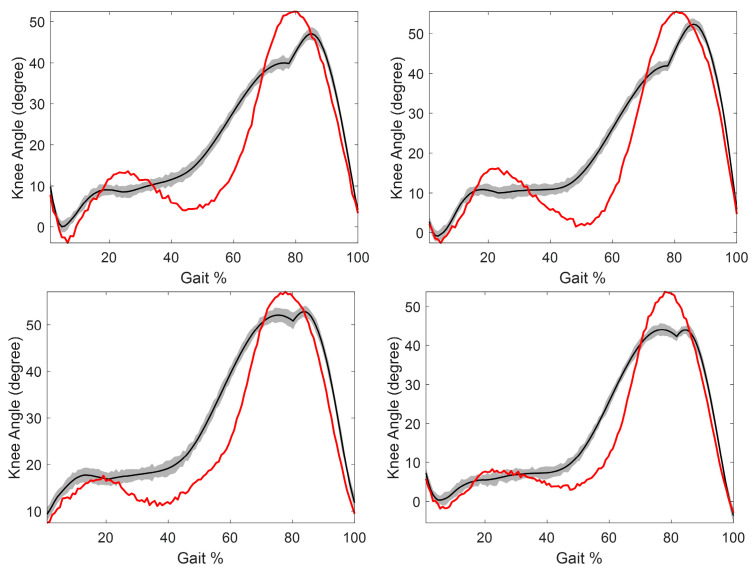
Exemplary strides from each of the four healthy volunteers showing the estimated knee angle (black) using GRU-AM model for each participant using 8 channels of sEMG. The shaded region is the 95% confidence region (mean ± 3 standard deviations) and red is the ground truth angle measured by motion capture.

An exemplary stride from each of the four healthy volunteers is shown in Figure 6 to demonstrate inter-participant performance. This visual depiction shows that the GRU-AM model consistently had the most accurate performance during the early stance and late swing phases. Also, the GRU-AM was able to estimate the knee extension during the swing phase with an overall similar range of motion as the ground truth; however, it had difficulty accurately estimating the peak knee flexion and extension angles for all participants.

**Figure 7:**
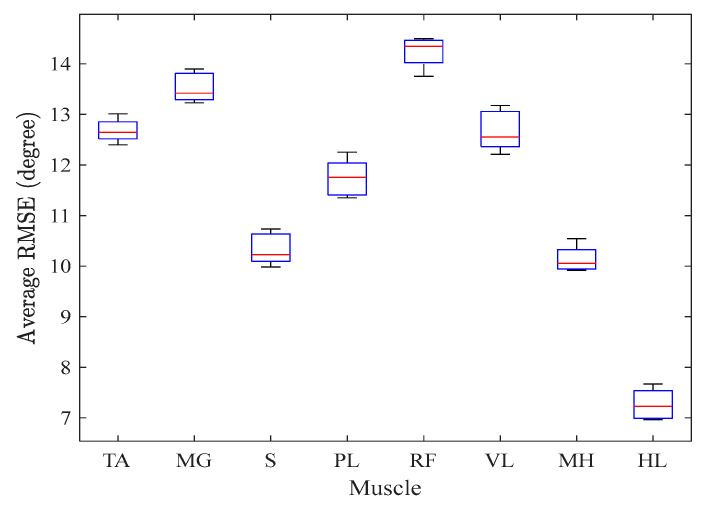
Sensitivity analysis of the GRU-AM knee angle estimator. In each boxplot, the median root mean squared error (RMSE) is depicted as a red line, capturing the central performance measure. Surrounding this, the upper and lower quartiles are presented, delineating the range and dispersion of RMSE values across the four healthy participants.

**Figure 8:**
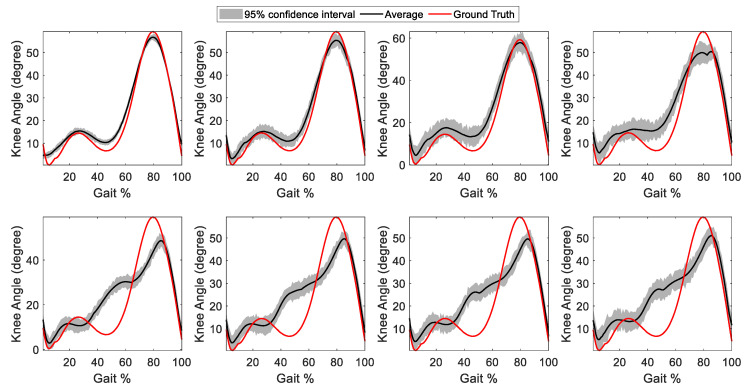
Exemplary data showing the effect of removing one sEMG channel on knee angle estimation for healthy participant 2. The muscles removed were top row, left to right: HL, PL, MH, SL and bottom row, left to right: RF, TA, MG, VL. In each plot, the shaded region indicates the 95% confidence region and the red line is the ground truth knee angle measured by motion capture. Knee angle estimation accuracy is most affected by the removal of RF, TA, MG and VL.

To further evaluate the GRU-AM sensitivity to the input muscle set, we removed each sEMG sequentially and computed the RMSE and CC. As depicted in Figure 7, the model’s accuracy demonstrates a distinct pattern as muscles are incrementally removed. The four muscles that resulted in the greatest increase in RMSE when removed were the RF, MG, TA and VL, indicating they were primary contributors to knee joint angle estimation from sEMG. A visual example of the effect of removing each sEMG channel from one healthy participant is shown in Figure 8. A key observation from Figure 8 is that the removal of single sEMG channels affects both the shape of individual stride estimation and the degree of noise present, particularly manifesting as high-frequency distortion in the output. Additionally, the removal of RF, TA, MG, and VL significantly impacts the mid- and late-stance phase estimate accuracy, as elucidated in the second row of Figure 8.

### 3.2. Experimental 2: Transfer Learning

The objective of this experiment was to investigate the impact of transfer learning. Initially, data from six sEMG channels, collected from four healthy participants in an open-source dataset, were employed to train the GRU-AM estimator for knee angle prediction. The primary aim was to leverage a pre-trained GRU-AM model from four healthy participants in the open-source dataset. The GRU-AM was personalized through initial training with 10 strides from each individual. Additionally, we aimed to establish a Cyclic Update Period, a crucial consideration given the computational complexity in real-time applications. We determined that a three-stride interval, equivalent to approximately 3 megabytes of memory resources, effectively balanced computational efficiency with the training data’s quantity. This strategic selection harmonized the update period with processing time and data volume, a critical factor for real-time learning. The estimated knee angle and associated performance metrics, namely RMSE and CC, were computed for each successive stride. This evaluation procedure is applied to four NIH participants, encompassing two trials for each participant where each trial consists of 30 strides, encompassing 10 learning cycles. Spatiotemporal data from the NIH participants are shown in Table 1.

**Figure 9:**
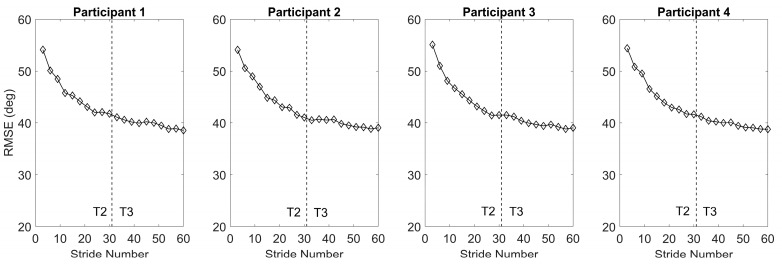
Intra-participant performance of the neural network with attention-based convergence performance of GRU-AM model tested by NIH dataset and applying cyclic training at the end of each 3 strides. The model was initially trained with an open source dataset and tested with a single NIH healthy participant. The first 30 strides are T2 and the second thirty are T3 as indicated.

The pretrained GRU-AM exhibits a notable pattern of gradual improvement in estimation performance across trials in all four participants (Figure 9). The term “average convergence rate” encapsulates this enhancement rate, representing the progressive enhancement in estimation performance with each cycle. The average convergence rate is the slope of the line that connects the first stride’s RMSE and the last stride’s RMSE. For all participants within the NIH dataset, the initial trial (T2) showcases an average convergence rate of −0.5 RMSE/stride. Notably, the third trial (T3) demonstrates a slightly slower rate of estimation enhancement (−0.2 RMSE/stride) compared to T2. This finding indicates that intra-participant performance improves through sequential learning, substantiating the efficacy of the approach. These outcomes underscore the potential of sequential learning to enhance the model’s estimation performance over consecutive cycles.

### 3.3. Experiment 3: Progressive Adaptation in Participants with CP

**Figure 10:**
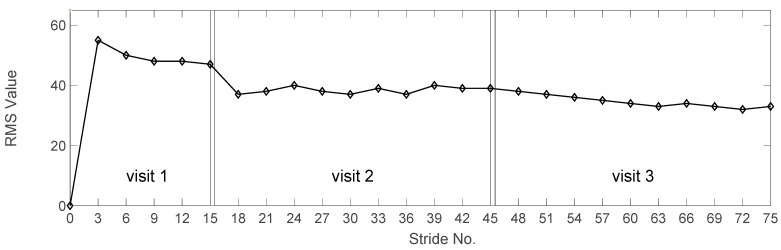
Estimation accuracy of a pretrained model with a healthy participant and tested with a participant with CP. The figures from left to right show the learning progression over three visits and sequential learning occurs at the end of three strides.

**Figure 11:**
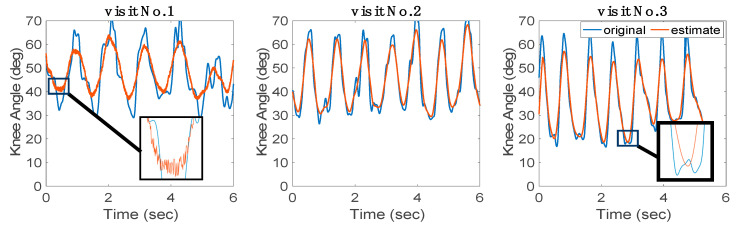
Learning progression of a pretrained model tested with a participant with CP. The sequence illustrates the model’s adaptation over three visits, with cyclic learning initiated at the conclusion of every three strides.

In this experiment, the GRU-AM model was initially trained with data from four participants from the open-source dataset and four healthy participants from the NIH dataset. Then, the GRU-AM was evaluated with data from the participant with CP in a sequential learning experiment whereby the model was updated every three strides. Figure 10 depicts the progressive performance of the knee angle estimation by recording the RMSE at the end of the three strides. The first visit of the CP participant comprises 15 strides, and the second and third visits comprise 30 strides. Using sequential learning to update the GRU-AM model reduced the RMSE by 20%, 16% and 15% within the first, second and third visits, respectively. This was true even as the gait speed increased progressively across visits (Table 1). The sEMG signals recorded from the participant with CP exhibited higher noise levels compared to healthy participants. This noise, coupled with the recurrent nature of GRUs and the presence of the attention mechanism, tends to impact the quality of the estimator’s output, as demonstrated in Figure 11 (left), resulting in greater RMSE in the first visit. However, the application of the sequential training policy contributes to noise reduction, as evidenced in Figure 10 and Figure 11 (middle and right). This phenomenon leads to a discernible improvement in estimator performance over the course of the three visits. The reduction in RMSE values across successive cycles highlights the estimator’s competence in estimating knee angle from sEMG despite the presence of an exoskeleton.

**Figure 12:**
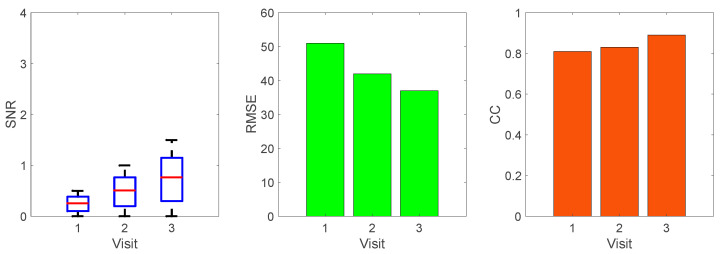
The average estimation performance metrics to estimate the knee angle signal of a participant with CP during the 1st, 2nd, and 3rd visit.

While it is challenging to precisely quantify the reasons behind noise reduction, the estimator’s overall performance significantly improves after the second visit. Figure 12 illustrates the average SNR across the three trials of each visit, showcasing an enhancement in SNR from visit to visit. Note that the SNR value increases in successive visits as the sequential training strategy leads to improvements in CC and RMSE (Figure 12).

**Figure 13:**
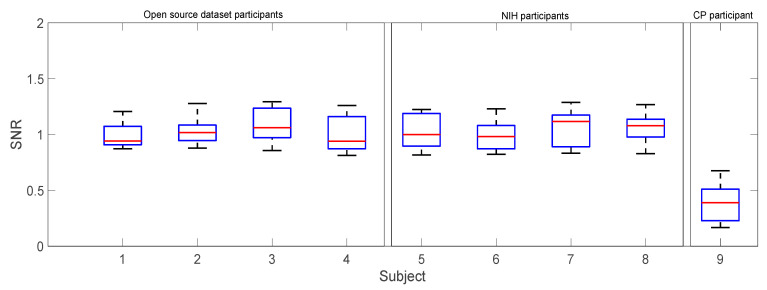
The average SNR in the resulting knee angle during trial 2 and trial 3 for the open source dataset and NIH participants including the participant with CP.

Our analysis of the SNR not only demonstrates differences between healthy participants and the child with CP but also sheds light on the intricacies of signal quality in knee angle estimation. Figure 13 depicts that the SNR of the GRU-AM knee angle estimate from healthy participants from the NIH dataset is consistently maintained from 0.99 to 1.6, signifying a relatively stable and clear signal. This finding underscores the consistency of signal quality in typical gait patterns across individuals. Similarly, healthy participants from the open-source dataset maintain SNR values within the range of 0.6 to 1.5. This result implies that the model retains its accuracy even with fewer strides per trial (10 rather than 30), indicating its ability to perform well in scenarios where data collection is more constrained.

In stark contrast, Figure 13 shows that the CP participant exhibits an average SNR of 0.41±0.16, which is substantially lower than healthy participants. This discrepancy highlights the considerable challenges associated with noise in CP-related knee angle estimations. The high noise levels encountered in the EMG from the participant with CP underscore the importance of developing robust algorithms capable of handling varying signal qualities, particularly in clinical contexts where such challenges are prevalent. These findings collectively underline the positive impact of sequential learning on the estimator’s performance metrics, illustrating its potential utility in real-world applications, particularly for a participant with CP undergoing rehabilitation.

## 4. Discussion

Our results demonstrate the ability to estimate knee angle during overground walking in healthy adolescents through the use of an RNN using GRUs and an attention mechanism. On average, the estimation improved with the attention mechanism compared to the same GRU without it, indicating its ability to focus on the most relevant parts of the inputs within each time window. In this case, this likely results in more heavily weighting muscles that are active during knee excursion in overground walking. The sensitivity analysis (Figure 7) corroborates this finding as the GRU-AM prediction is most sensitive to the knee extensor and flexor muscles. Our results also underscore the model’s potential to generalize knowledge across diverse datasets via transfer learning, as a model that was trained on open source biomechanics data from healthy adults was successfully deployed to estimate knee angle during walking in healthy adolescents. Further, the sequential learning—in which the model was successively trained over three stride periods—showed continued improvement in knee angle estimation (Figure 9), demonstrating the attention-based estimator model’s adaptability and robustness in knee angle estimation over time, supporting its eventual use in real-world scenarios.

The third experiment demonstrates successful transfer and sequential learning to estimate knee joint angle during overground walking in a child with cerebral palsy (CP) while an exoskeleton was worn. Importantly, this participant was assessed clinically with mild-to-moderate spasticity of the knee extensor and flexor muscles and functioning at GMFCS Level III. The GRU-AM approach was capable of estimating knee angle with a reasonable accuracy, despite these alterations in neuromuscular functioning. It should be noted that the estimator accuracy was lower in the child with CP compared to the healthy individuals, a result that is not surprising given the difference in function.

There have been multiple prior studies that have demonstrated the ability to estimate lower limb kinematics during walking from EMG using formulations that include attention mechanisms (AM), gated recurrent units (GRU), and/or transfer learning LSTM approaches [35,36,50]. These studies include walking, running, as well as squatting and sit-to-stand and stand-to-sit maneuvers. Notably, these studies all were performed on healthy individuals. As in these prior studies, normalized RMSE (NRMSE), which is computed by dividing the RMSE by the range of the measured (true) estimated outcome, can be used to compare estimation accuracy. By this metric, the findings from our first experiment, involving healthy adolescents, show improved NRMSE (3.5%) compared to these prior studies which report NMSRE from 6 to 10% [35,36,50]. Although these results suggest the novel GRU-AM formulation presented here may improve knee angle estimation from sEMG compared to prior approaches, it is important to note that the activities and outcome measures estimated from sEMG were not identical across these studies. Therefore, future investigations are needed to further evaluate the effect of such parameters, in particular the number of sensors, on estimation accuracy in both healthy and clinical populations. There are several other limitations that warrant discussion. First, this study included relatively short bouts of walking, with convergence examined across a maximum of 60 strides (in healthy individuals) and 30 strides (in CP); therefore, the ability of the GRU-AM to maintain stable performance across longer durations remains unknown. Similarly, the training update window consisted of three strides, which provided acceptable accuracy to demonstrate feasibility, but the duration of this window and its effect on convergence and stability should be examined in future work. Finally, the evaluation in the clinical population was limited to one individual; therefore, caution is necessary when extending these findings to the broader CP population. Additional experiments and evaluation are necessary to confirm the ability of the GRU-AM to decode kinematics from EMG in this and other clinical populations.

The findings here also fill crucial gaps in the existing literature on joint angle estimation from sEMG. First, we demonstrate that an individual-specific model can maintain, and even improve, joint angle estimation accuracy across multiple sessions/days. Second, we show that such estimation models are deployable in an individual with a movement disorder (CP), in which sEMG has a lower signal-to-noise ratio (SNR) and less stride-to-stride reliability than in healthy individuals. It is important to note that while our results support the feasibility of using the GRU-AM for each of these transfer learning tasks, we did not directly compare our method with others; therefore, future investigations should examine the ability of other ML algorithms in similar transfer learning tasks. Third, we verify that joint angle estimation can be performed from sEMG in the presence of an exoskeleton. Collectively, these findings suggest the potential for deployment of sEMG-based joint angle estimators as a method for inferring movement intent in individuals with movement disorders and provide an impetus for continued development toward deployment in real-time and real-world settings.

## Figures and Tables

**Table 1:** Spatiotemporal metrics.

		Number of Strides	Step Length ^1^ (m)	Gait Speed (m/s)
HV	1	60 ^2^	0.52 ± 0.02	0.91 ± 0.04
	2	60	0.49 ± 0.02	0.88 ± 0.03
	3	60	0.51 ± 0.01	0.87 ± 0.02
	4	60	0.56 ± 0.02	1.00 ± 0.05
CP ^3^	V1	15	0.20 ± 0.04	0.14 ± 0.03
	V2	30	0.19 ± 0.02	0.37 ± 0.16
	V3	30	0.17 ± 0.04	0.52 ± 0.06

^1^ Mean ± SD; ^2^ Data from each participant represented a random subsample of 60 strides; ^3^ Metrics were computed for each visit for the participant with CP.

## Data Availability

The data presented in this study are available on request from the corresponding author.
